# Electrocardiographic semi-spiked helmet sign in critically Ill patients: A case series

**DOI:** 10.1097/MD.0000000000035661

**Published:** 2023-10-27

**Authors:** Bryan Richard Sasmita, Suxin Luo, Bi Huang

**Affiliations:** a Department of Cardiology, the First Affiliated Hospital of Chongqing Medical University, Chongqing, China.

**Keywords:** case reports, coronary spasm, myocardial injury, sepsis, ST-segment elevation

## Abstract

**Rationale:**

ST-segment elevation on electrocardiogram (ECG) is an alarming sign. Although acute myocardial infarction (AMI) is the most common cause of ST-segment elevation, many non-ischemic conditions may produce pseudo-ST segment elevation. Spiked Helmet (SH) sign is one of the pseudo-ST segment elevations that is associated with critical illness and high risk of death. SH sign was characterized by an upward shift starting before the onset of the QRS complex; however, we found some patients presented with a peculiar characteristic on ECG with an upward convex ST-segment elevation after the QRS wave but without elevation before the QRS wave, therefore called Semi-SH sign. Also, this electrocardiographic feature exists in patients with critical disease and is related to poor prognosis. The purpose of this case series is to describe the electrocardiographic Semi-SH sign and enhance the awareness of such electrocardiographic manifestation for clinicians.

**Patients Concerns:**

This case series explores the possibility of severe infection induced electrocardiographic changes resembling spiked-helmet sign.

**Diagnoses:**

Sepsis-induced secondary myocardial injury or coronary vasospasm.

**Interventions:**

Gastric decompression, antibiotics, diuretics, advanced life support.

**Outcomes:**

The outcome of this case series is the association of the electrocardiographic Semi-SH sign with the prognosis. All 3 patients died several days post manifestation of electrocardiographic Semi-SH sign.

**Lesson:**

Like SH sign, electrocardiographic Semi-SH sign is a life-threatening or deadly ECG sign, and therefore early recognition and aggressive treatment are important.

## 1. Introduction

Acute myocardial infarction (AMI) often causes ST-segment elevation on electrocardiogram (ECG); however, many other diseases are also associated with ST-segment elevation, among which severe infection is a common cause. Meanwhile, this morbidity can also cause myocardial injury, resulting in an increased troponin level that resembles AMI. In 2011, Littmann et al^[1]^ reported 5 septic patients who had no typical manifestation of AMI presented with a dome-and-spike QRS complex resembling ST-segment elevation. These ECG change resembles a German military spiked helmet, Pickelhaube, hence known as Spiked Helmet (SH) sign.^[1]^ The SH sign was characterized by an upward shift starting before the onset of the QRS complex. However, we recently found some patients presented with a semi-SH sign that is an upward convex ST-segment elevation but without elevation before the QRS complex. This ECG presentation has distinct morphological features compared with the classic SH sign. Here we described several cases which presented with the semi-SH sign, and the prognosis of these patients was poor. Therefore, we should be vigilant and aware of such ECG manifestations in critically ill patients. Ethical approval for this study was obtained from the Ethics Committee of the First Affiliated Hospital of Chongqing Medical University. The written informed consent was obtained from the relatives of the patients.

## 2. Case series

### 2.1. Case 1

An 81-year-old man was admitted due to the recurrence of gastric cancer. One year ago, he was diagnosed with pathological stage III B (pT4aN2M0) gastric adenocarcinoma and underwent radical gastrectomy. Four months before, he underwent gastric endoscopy and abdominal CT scan, which showed a recurrence of gastric cancer with metastasis to the liver. Then he received combined chemotherapy and a trans-arterial chemoembolization procedure. This time he was scheduled to receive another gastrectomy and readmitted. After surgery, he received routine gastrointestinal decompression. Sixteen days later, the patient suddenly presented with a high fever (39.2˚C) and dyspnea. Inflammatory markers revealed elevated leukocyte 20.53 × 10^9^/L (normal range 3.5–9.5 × 10^9^/L), neutrophil 83.9% (normal range 40.0–75.0%), procalcitonin 45 ng/mL (normal range <0.05 ng/mL), interleukin-6 740.79 pg/mL (normal range < 5.40 pg/mL), and C-reactive protein 177.0 mg/L (normal range < 8.0 mg/L). Subsequently, the blood cultures were positive for methicillin-resistant staphylococcus aureus infection, thus, methicillin-resistant staphylococcus aureus-induced sepsis was diagnosed. In addition, antimicrobial resistance genes acting were found against beta-lactam antibiotics, macrolides, quinolones, and glycopeptides. Therefore, a renal-adjusted dose of imipenem, teicoplanin, and hydrocortisone was initiated.

The patient condition rapidly deteriorated and developed into septic shock. An ECG revealed an ST-segment change in the precordial leads with an upward convex ST-segment elevation after a sharp R wave with a notch between the R wave and ST segment. Biomarkers of myocardial injury indicated cardiac troponin I at 1.1 ng/mL (normal range < 0.023 ng/mL), creatinine kinase MB at 4.2 ng/mL (normal range 2–7.2 ng/mL), and myoglobin at 900ng/ml (normal range 23–112 ng/mL). Bedside echocardiography revealed diffused left ventricular hypokinesis with an ejection fraction of 35% (normal range 55%–75%); subsequent radiology did not find coronary artery stenosis, pulmonary embolism, or intestinal obstruction. In addition, the patient did not complain of chest pain or other angina-equivalent symptoms during the whole period. Although the patient received aggressive treatment, including tracheostomy, gastrointestinal decompression, and antibiotic combination therapy, he gradually developed multiple-organ failure and died 2 weeks after the electrocardiographic change.

### 2.2. Case 2

A 61-year-old man was admitted due to intrahepatic cholangiocarcinoma. One week prior to admission, the patient presented with abdominal distention, jaundice, and weakness. CT scan showed a mass in the liver and left lung. Subsequently, a biopsy was performed, and metastatic intrahepatic cholangiocarcinoma was diagnosed. Upon admission, he presented with jaundice, tachycardia (110 beats/min), tachypnea (22/min), and elevated blood pressure (150/84 mm Hg). He had no history of cardiovascular diseases. The patient was given gastric decompression, hepatoprotective drugs, diuretics, propranolol, and somatostatin. Eleven days post-admission, the patient suddenly suffered a cardiac arrest. Resuscitation with advanced life support, including intubation and repeated epinephrine was administered. Unfortunately, after resuscitation, the patient still went into a comatose state. On the following day, the patient presented with a high fever (39.9°C), tachycardia (119 beats/min), and hypotension (90/63 mm Hg). A blood routine examination showed that leukocyte was elevated at 19.42 × 10*9/L (normal range 3.5–9.5 × 10^9^/L). Blood coagulation test showed prothrombin time 37.0s (normal range 11.0–14.5s), international normalized ratio 3.73 (normal range 0.80–1.20), activated partial thromboplastin clotting time 111.5s (normal range 28.0–44.0s), thrombin time 76.5s (normal range 14.0–21.0s), and D-dimer 76 mg/L (normal range 0.2–0.7 mg/L). Biomarkers of myocardial injury indicated creatinine kinase MB was elevated at 2.1 ng/mL (normal range 2–7.2 ng/mL), and troponin T was elevated at 0.203 ng/mL (normal range < 0.030 ng/mL). Echocardiography revealed normal left ventricular systolic and diastolic function, while ECG indicated an upward convex ST-segment elevation in the precordial leads. The patient presented with multiple organ dysfunction and underwent several episodes of ventricular tachycardia, and finally, he died 5 hours after the electrocardiographic change.

### 2.3. Case 3

A 79-year-old woman was admitted with dizziness and bilateral leg weakness for 11 hours. She had a history of hypertension, diabetes mellitus, coronary heart disease, hyperthyroidism, and atrial fibrillation. On admission, the patient was hemodynamically stable with a blood pressure of 127/63 mm Hg, and a heart rate of 54 bpm. Neurological examination revealed that her pupils were normal and equally reactive to light, however, she had a speech disturbance, muscle weakness, and positive Babinski sign on both legs. Emergency head CT showed an infarction on the temporal and parietal lobes. The patient was given clopidogrel, atorvastatin, anti-hypertensive, and anti-diabetic drugs during hospitalization. Twenty-three days later, the patient suddenly developed abdominal pain in the right iliac fossa. Abdominal examination revealed tenderness and rebound tenderness in McBurney point, along with the absence of peristalsis. Arterial blood gas analysis showed pH 7.50 (normal range 7.35–7.45), PCO_2_ 20 mm Hg (normal range 35–45 mm Hg), PO_2_ 109 mm Hg (normal range 75–100 mm Hg), and lactate 6.4 mmol/L (normal range 0.5–1.5mmol/L). Blood examination showed leukocyte was significantly elevated at 30.63 × 10*9/L (normal range 3.5–9.5 × 10^9^/L) with neutrophil 92.3% (normal range 40.0%–75.0%). Biomarkers of myocardial injury indicated an elevation of troponin T at 0.750 ng/mL (normal range <0.1 ng/mL). Echocardiography showed an enlarged left and right atrium with normal systolic function (left ventricular ejection fraction 64%). Abdominal CT suggested acute appendicitis, thus appendectomy was performed. Three days after the operation, the patient suddenly developed circulatory failure with multiple organ dysfunction. An ECG demonstrated atrial fibrillation with ST-segment elevation in the anterior leads. One day later, this patient died of multiple organ failure.

## 3. Discussion

**Figure 1.**
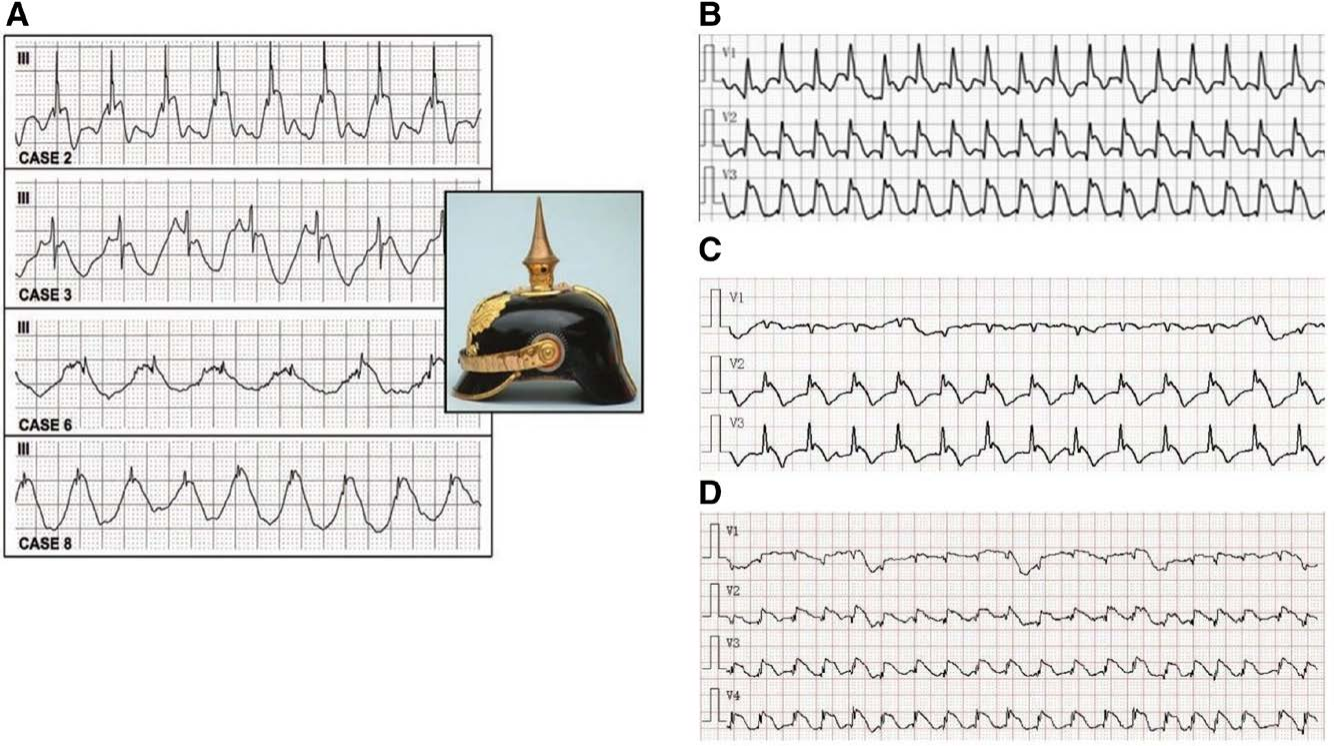
The comparison of the classic SH sign with semi-SH sign. (A) Classic SH sign (Littmann et al), the upward shift starting before the onset of the QRS complex. (B, C, and D) Representative electrocardiograms of 3 patients showing the semi-SH sign, an upward convex ST-segment elevation after a sharp R-wave with the section before QRS complex was at the baseline level. Pictures obtained the copyright permission of the corresponding publishers. SH = spiked helmet.

The classic electrocardiographic SH sign described in Littmann et al’s^[1]^ study was characterized by a dome- and spike-like ST-segment elevation. Meanwhile, in addition to ST-segment elevation, the P-R interval preceding the QRS wave was also elevated, nearly symmetric as a German military spiked helmet, the so-called SH sign. However, our present study found a similar but distinct morphology compared with the electrocardiographic SH sign, and we called it a semi-SH sign. The major difference between the semi-SH sign and SH sign was that in the semi-SH sign, the section before the QRS complex wave was at the baseline level, while in Littmann et al’s^[1]^ study, both P-R interval and ST-segment were elevated (Fig. 1).

Initially, it was postulated that the SH pattern was associated with increased intrathoracic or intra-abdominal pressure,^[1]^ which directly stimulated the diaphragm affecting the inferior wall of the left ventricle or triggering the left leaf of the diaphragm through the left phrenic nerve, finally resulting in alteration of the ST segment.^[2]^ However, a subsequent study revealed a series of diseases, such as subarachnoid hemorrhage, sepsis, and respiratory distress that did not cause elevated intra-thoracic or intra-abdominal pressure, could also present with SH sign on ECG.^[1]^

In Littmann et al’s initial report,^[1]^ the SH sign was more prevalent in the inferior leads; however, in our case series, most of the semi-SH pattern was located in anterior leads (V2 and V3). The precise mechanisms of the different locations of SH sign on ECG remain unclear. According to the current reports, acute abdominal events were usually associated with the SH sign in inferior leads, whereas acute intrathoracic events tended to cause SH sign in the chest leads. In our case series, all patients received routine gastrointestinal decompression. Moreover, physical examination revealed no apparent signs of intestinal obstruction or tympanites. Therefore, the electrocardiographic semi-SH sign seems unrelated to the primary disease. Furthermore, the semi-SH sign occurred when the patient condition worsened. In fact, most cases in Littmann, et al’s^[1]^ report had a severe infection, especially sepsis. Similarly, in our present case series, all had severe sepsis. Hence, we inferred that the electrocardiographic semi-SH sign might result from infection induced myocardial injury in these cases.

Several findings may support our hypothesis. First, echocardiography did not present with segmental hypokinesis because coronary artery-related hypokinesis was usually specifically located in the area associated with the occluded artery. Second, most previous cases were reported to have negative cardiac troponin, whereas in our case series, all patients had elevated cardiac troponin; however, the extent of elevation was not as high as that in patients with AMI. This phenomenon indicated a moderate and transient myocardial injury rather than AMI because the cardiac troponin level did not undergo significant dynamic changes as AMI. Third, one of these patients received pulmonary and coronary CT angiography and excluded the thrombosis. Based on these findings, sepsis-induced secondary myocardial injury or coronary vasospasm could best explain this electrocardiographic phenomenon (Table 1).

**Table 1:** Clinical characteristics of patients with “Semi-Spiked Helmet Sign”.

Features	Case 1	Case 2	Case 3
Age	81	61	79
Race	Asian	Asian	Asian
Gender	Male	Male	Female
Main diagnosis	Sepsis	Metastatic Intrahepatic Cholangiocarcinoma, Sepsis	Sepsis
Interventions	I/MV, ALS, GD, antibiotics, gastrectomy, tracheostomy.	I/MV, cardioversion, ALS, GD, antibiotics, diuretics, somatostatin, propranolol, hepatoprotective drugs.	I/MV, ALS, antibiotics, GD, appendectomy, antiplatelets, anti-diabetic.
Semi-spiked helmet sign	V2 and V3	V2 and V3	V2 to V4
Admission to ECG manifestation (d)	16	11	25
ECG manifestation to death	14	1	1

ECG = electrocardiogram.

The mechanism behind sepsis-induced secondary myocardial dysfunction is not well understood, with several theories having been proposed, such as oxygen supply-demand imbalance,^[3]^ changes in coronary microcirculation,^[4]^ and downregulation of beta-adrenergic receptors and depression of post-receptor signaling pathway.^[5]^ Moreover, approximately 50% of patients with severe sepsis and septic shock may develop ventricular dysfunction, and cardiac troponin elevations correlate with left ventricular systolic dysfunction.^[6]^ Sepsis-induced ST-segment elevation has been reported in a select number of cases and is mostly presented with normal coronary arteries. Hence, coronary artery vasospasm, regional myopericarditis, and low coronary perfusion were discussed as potential mechanisms.^[1,7,8]^

The most important differential diagnosis of SH sign is ST-elevation myocardial infarction. However, patients presenting with SH sign often lack typical chest pain symptoms, and cardiac troponin is usually within the normal range or slightly elevated. Moreover, these patients may have underlying diseases such as pulmonary, abdominal, severely infectious, or cerebral diseases, which may cause myocardial injury. Furthermore, this electrocardiographic change can resolve after the primary disease is improved. Nevertheless, further examination, such as coronary angiography should be considered in specific patients to avoid misdiagnosis.

Although the electrocardiographic SH sign is temporary and reversible, it can be classified as a deadly or life-threatening ECG finding. For example, in Littmann et al’s^[1]^ study, 6 out of 8 patients who presented with an SH sign died after the initial presentation of the SH sign (mean 5.5 days). Thus, the SH sign was regarded as a new electrocardiographic marker of critical illness. In fact, in the present case series, all patients died after the manifestation of the semi-SH sign (mean 5 days), consistent with the conclusion from Littmann et al’s^[1]^ study.

There are some limitations in the present case series. First, due to the low incidence of electrocardiographic Semi-SH sign, we only reported 3 cases with such electrocardiographic presentation. More cases should further confirm this rare electrocardiographic feature. Second, although this electrocardiographic presentation is usually related to non-cardiac diseases such as severe infection, the sensitivity and specificity of this electrocardiographic presentation are not well understood. In addition, we hypothesize sepsis-induced myocardial injury or coronary vasospasm as the main mechanism of electrocardiographic semi-SH sign; however, the precise mechanisms are still unclear. Therefore, more studies are warranted.

## 4. Conclusion

To conclude, like a classic SH sign, an electrocardiographic Semi-SH sign is associated with a poor prognosis, and awareness of such electrocardiographic manifestation is essential for clinicians.

## Author contributions

**Conceptualization:** Bryan Richard Sasmita, Suxin Luo, Bi Huang.

**Data curation:** Bryan Richard Sasmita, Bi Huang.

**Formal analysis:** Bryan Richard Sasmita, Bi Huang.

**Supervision:** Suxin Luo.

**Validation:** Bi Huang.

**Writing – original draft:** Bryan Richard Sasmita, Bi Huang.

**Writing – review & editing:** Bryan Richard Sasmita, Bi Huang.
